# Strengthening Transversus Abdominis in Pregnancy Related Pelvic Pain: The Pressure Biofeedback Stabilization Training

**DOI:** 10.5539/gjhs.v4n4p55

**Published:** 2012-05-28

**Authors:** Dharmarajan Rajalakshmi, N. Sundaramurthy Senthil Kumar

**Affiliations:** 1 Faculty of Therapeutic Sciences, Masterskill University College of Health Sciences, Cheras, Malaysia

**Keywords:** pregnancy related pelvic pain, transverse abdominis, pressure biofeedback unit, efficacy, pain, disability

## Abstract

Pregnancy related pelvic pain (PRPP) refers to musculoskeletal type of persistent posterior pelvic pain during and after pregnancy with feature of reduced endurance capacity for standing, walking and sitting which leads to severe discomfort and considerable impairment of daily activities.

**Objective:**

To test the effect of pressure biofeedback stabilizer training, on the pain and dysfunction of a thirty year old subject who presented with PRPP.

**Study design:**

Single case design. Outcome variables: Oswestry pain and disability index, TrA efficacy.

**Methodology:**

An initial assessment was followed by treatment sessions which consist of 2 phases (Phase A & Phase B). The baseline phase (A) consists of conventional therapeutic exercises while the intervention phase (B) consists of pressure biofeedback training in conjunction with the conventional therapeutic exercises.

**Result:**

The study data demonstrated that the subject showed minimal improvement in pain, disability and TrA efficacy during the baseline phase and shown a steady improvement in all these variables during the intervention phase.

**Conclusion:**

Core muscle performance (TrA) can be retrained with pressure biofeedback stabilization training program in subject with PRPP thereby reducing pain and disability.

## 1. Introduction

Evidence is growing that Pregnancy related pelvic pain (PRPP) is a distinct clinical entity, the exact causes of which still remain unknown (O’Sullivan & Beales, 2007). Previous literature suggests that around half of all pregnant women incur PRPP (Rungee, 1993; Berg, Hammar, Möller-Nielsen, Lindén & Thorblad, 1988; Fast et al., 1987) and in some patients it leads to severe disability (Wergeland & Strand, 1998; Ostgaard, Andersson, & Karlsson, 1991). In recent literatures the prevalence rate ranges from 4% to 90% with 50% median and nearly10-75% of the women had persisting pain for 1-3 months after delivery (Van De Pol et al., 2007; Mousavi et al., 2007; Bastiaenen et al., 2006; Gutke et al., 2006). There were many research studies dedicated to this topic during the last two decades. PRPP has been a vague terminology for a long time. The variety of terms used to describe the condition, and the unclear diagnostic criteria could probably influence the prevalence rate.

PRPP was broadly defined as a general symptom of persistent pain located in the pelvis, of at least several months’ duration. Ostgaard, Zetherström, Roos-Hansson, Svanberg (1994) proposed, “posterior pelvic pain” to denote problems that are distinct from low back pain in pregnancy, and it is not yet completely understood how functional pelvic impairment relates to anatomical structures. Larsen et al. (1999) mentioned that in most patients, the symptoms ceased shortly after delivery. However, in 4% of the patients, the symptoms persisted over several months postpartum. Wu et al. (2004) demonstrated that women with PRPP have difficulty in walking and were often unable to cover large distances and, the most problematic activities were housework activities with the children, employment, leisure/hobbies, exercise and personal relationships or married life. Possible causal factors in Ostgaard et al. (1991) study, indicates previous pelvic girdle pain as a strong risk factor, not only with respect to the occurrence of pain but also with respect to pain intensity and the duration of pain in a future pregnancy. In addition, multiparity was moderate important to the risk of having PRPP but was an important factor in determining the length of the pain period and was related to pain intensity late in pregnancy.

The earliest explanation came from Hippocrates (460-377 B.C). His theory was that an irreversible relaxation and widening of the pelvis occur with the first pregnancy that resultant instability of the sacroiliac joints leading to symptomatic inflammation (Lynch, 1920). Since that time, pregnancy-related pain in the pelvis has been recognized as severe and disabling. During 1940s to 1990s, most of the study supported that relaxation of the pelvic joint cause pelvic insufficiency during pregnancy and after parturition. Hansen et al. (2005) pointed out that pelvic pain during pregnancy causes considerable impairment of daily functions and was termed symptom-giving pelvic girdle relaxation (PGR) in pregnancy. Most hypotheses focused on changed load resulting from increased weight and decreased stability of the pelvic girdle because of hormonal changes. PRPP occurs during pregnancy or within first three weeks after delivery and was found to be higher in second and later pregnancies (Endresen, 1955).

Mens et al. (2009) compared the patients who suffer from pelvic girdle pain with healthy pregnant women and established that the patients with pelvic girdle pain have increased motion in their pelvic joints, which results in negative consequences namely decrease in the efficiency of load transmission and increase in the shear forces across the joints. There is a possibility that these increased shear forces are responsible for pain in pregnant women with pelvic girdle pain (Vleeming et al., 2008; Vermani et al., 2010)

Successful diagnosis and the effective therapy depend on early interpretation of symptoms and the outcome of the diagnostic process. Available literature argues that, the intervention program for this group of patients aims at providing adequate information and reassurance, to reduce fear. And were encouraged to do specific exercises with adequate rest in between and were informed to be active, but not to overload the pelvis. PRPP can be seen differently between professions, chiropractors often use the term sacro-iliac joint, SIJ, malfunction or SIJ position while physiotherapists have a biomedical perspective in terms of inflammation or weakness (Vermani et al., 2010). The therapist set the goal of treatment based on intensity and duration of reported pain and they often include specific stabilization exercises to overcome the muscle imbalance and the malalignment of pelvic girdle. There is dearth of studies in the treatment of pelvic pain attributed to pregnancy and child birth, of which one study focus on specific stabilizing exercises with a positive result (Young & Jewell, 2002). This suggests uncertainty about an optimal approach. Here we report one patient in whom back pain was erroneously attributed to pregnancy and childbirth. In this study, we use pressure biofeedback unit (PBU) as an intervention to work on back stabilizers. It is a reliable and valid clinical instrument for assessing deep abdominal muscle function, and has been used in various studies to develop a method for the careful monitoring of spinal stabilization (Cairns, Harrison, & Wright, 2000; Jull & Janda, 1987). The objective of this case study was to test the effect of pressure biofeedback stabilizer training, on the pain and dysfunction of a thirty year old patient who presented with PRPP.

## 2. Research Methods

### 2.1 Case History

A woman aged 30 years was referred for physiotherapy by her General Practitioner, 2 weeks after her third uncomplicated pregnancy and delivery with history of low back pain. The pain was aggravated when she lifted her baby but had been getting progressively worse with pain radiating to the left side. Patient reports to hospital when she developed severe back pain.

On admission the clinical symptoms were stabbing pain in the SI joint (PN1), pain was rated 9/10 on the numeric visual analogue scale (VAS). After the admission the patient had mostly bed rest for two days. Pain eased to 8/10 on the VAS post admission. The pain was described as a stabbing sensation which aggravates on prolonged standing, sitting, carrying and lifting her baby. A cold pack on her back helped to reduce pain temporarily. The patient also experienced radiating pain (PN2) on the left glutei and posterior thigh rated 3/10 on the VAS.

The patient reported previous episodes of low back pain during her pregnancy, but only now and never as severe as at the time of the assessment. The patient was also concerned that she has never experienced PN2 in the past. The patient was on paracetamol to manage pain, as she was feeding her baby. The patient’s activities of daily living were affected since she experienced severe pain with feeding and carrying her baby for frequent periods of time. The patient presented with an antalgic gait with reduced walking speed.

Pain on initial physiotherapy assessment was 8/10 in the SI joint and 3/10 in PN2 area. The clinical examination included a mechanical assessment of the lumbar spine (McKenzie protocol) to identify subject likely to have discogenic pain. The lumbar mechanical assessment consist of flexion, lateral flexion and extension in standing, flexion in lying (knees to chest), extension in lying (a half press-up with the pelvis remaining in contact with the table). The test movements were repeated in sets of 10 and there was no centralisation or peripheralisation phenomena observed, hence the clinical decision that symptoms were produced by disc pathology was ruled out to be negative.

The Thigh thrust, Gaenslen’s test, Sacral thrust and Patrick’s Faber test were positive and the Modified Trendelenburg’s test was negative, hence it is evident that the pathology is at the SI joint (Ostgaard, Zetherström, & Roos, 1994; Vleeming, de Vries, Mens, & Van, 2002).

As a functional test, ASLR is performed in supine lying with feet 20cm apart. Without bending the knee the subject is asked to raise one leg 20 cm above the examining table and her feeling of impairment was scored on a 6-point scale: not difficult at all = 0; minimally difficult = 1; somewhat difficult = 2; fairly difficult = 3; very difficult = 4; unable to do = 5 (Craig et al., 2009; Mens, Vleeming, Snijders, Koes, & Stam, 2001 & 2002). The scores of both sides are added and a total score obtained was 4 which was considered positive and did not reproduce PN1 or PN2.

On soft tissue palpation, tenderness was graded as 3 and 1 at left and right long dorsal ligament respectively. All other accessory movements of the lumbar spine were within normal limits.

From the findings on the subjective and objective examination, it was confirmed that the patient’s pain was caused by muscular imbalance and it is purely a SI joint mechanical cause.

Progress was measured with the Oswestry pain and disability index and TrA efficacy.

### 2.2 Study Design

A single case design was used, and the study was blinded to minimize the investigator bias.

### 2.3 Instruments

Chattanooga stabilizer pressure biofeedback sensor and a stopwatch were used to measure the change in pressure during TrA contraction. PBU provides a basic, valid and inexpensive measurement tool for the purposes of assessment, monitoring and feedback (Richardson, Jull, Hodges, & Hides, 1999) (Cole, Finch, Gowland, & Mayo, 1994; Hodges & Richardson, 1996 & 1997; Cairns et al., 2000). The PBU test has been validated by imaging and electromyography, tests that are considered to be the gold standard measurements of TrA performance (Teyhen et al., 2007, 2008). The pressure biofeedback unit consists of an inflatable cushion (pressure cell) connected to a pressure gauge and an inflation bulb. It is a simple device which registers changing pressure in an air filled pressure cell. This device provides feedback to ensure quality and precision in exercise performance and testing thereby improve back health.

### 2.4 Procedure

On the first visit, the subject underwent a standardized musculoskeletal examination. The outcome measures were Modified Oswestry low back pain disability questionnaire, and TrA efficacy using PBU. The assessment was followed by treatment sessions which consist of 2 phases (Phase A & Phase B). The subject attended physical therapy thrice a week (4 days rest in order to avoid over stressing) for 6 weeks (18 visits), which includes 3 weeks (9 visits) of baseline (A) phase and 3 weeks (9 visits) of intervention (B) phase. The baseline phase consists of conventional therapeutic exercises while the intervention phase consists of pressure biofeedback training in conjunction with the conventional therapeutic exercises. The exercises were designed for reducing pain, core strengthening, and improving functional activity. The outcomes measures were obtained at the end of each phase.

To calculate efficiency and to train the TrA muscle, the basic concept is an isolated action of the TrA muscles. Abdominal drawing in maneuver (ADIM) is taught by asking the subject to gently draw in the lower abdominals (Richardson & Jull, 1995) and the subject was asked to hold the contraction for 10 seconds. Initially she was doing more of breath holding and rib elevation. Initial training includes tactile cueing in which the subjects were asked to palpate, 1 inch medial and inferior to the anterior superior iliac spine (ASIS) to confirm the contraction of the TrA. Previous studies have proved that in this maneuver, forward drift of abdominal contents results in a facilitatory stretch of the deep abdominal muscles (Richardson, Jull, Toppenberg, & Comerford, 1992; Beith, Synnott & Newman, 2001; Norris, 2001) and that the EMG activity reveals an inhibitory activity of global muscle, rectus abdominis (Richardson et al., 1995). The subject was asked to practice ADIM not more than 6 times inorder to prevent premature fatigue (Cairns et al, 2000).

For TrA training the subject was position in crook lying, the pressure cell was placed under the lumbar spine and was inflated to the baseline pressure of 40mm Hg. The subject was asked to selectively contract her TrA (the baseline pressure should remain stable) without moving her spine and pelvis and asked to hold for 10 seconds, over 4 set (1 set=10 consecutive contraction) and progressed by one set every week as suggested by Ferreira et al (2009) and Teyhen et al (2005).

### 2.5 Outcome Measures

An Oswestry questionnaire consists of 10 sections. Each section scored from 0 to 5 with higher values indicating more severe impact. The result is expressed on a scale ranging from 0 (no pain or difficulties) to 50 (highest score for pain and greatest difficulty on all items). Disability in percent = (total score)/50 × 100(0% to 20%- minimal disability, 21% to 40%- moderate disability, 41% to 60% -severe disability, 61% to 80% -crippled, 81% to 100% -bed bound).

The subject was position in prone lying, to measure anterolateral abdominal function (TrA efficacy) as this position has been reported to have an apparent inhibitory effect on rectus abdominis (Richardson et al., 1995, Beith et al., 2001). The inflatable cell was placed beneath the abdomen with the lower edge at the level of the anterior superior iliac spine (ASIS) and was inflated to the baseline of 70 mm Hg, and the subject was instructed to contract her TrA selectively. Pressure should decrease 6-10 mmHg from the baseline. Readings were taken from the start and finish of 10 second contraction over ten consecutive contraction (timed using a stopwatch). The PBU was zeroed to 70 mmHg (baseline) before each contraction, changes in pressure and its mean were calculated at the end of ten contractions. During data collection no feedback was given to the patient.

### 2.6 Data Analysis

The outcome measures were compared between initial, post baseline and post intervention phases. Initially Oswestry was scored 48 indicating 96% disability and she was bed bound and the mean change in pressure which is a measure of TrA efficacy was scored minus 2. The post baseline assessment following Phase A (Conventional exercise), Oswestry was scored 35 with 70% disability with a mean change in pressure was scored as minus 5 and the post intervention assessment following Phase B (PBU training with Conventional exercise) Oswestry was scored as 6 with 12% disability with a mean change in pressure as minus 8.

## 3. Discussion

The aim of the study was to study the effect of PBU on pain and disability in subject with PRPP.

The PBU test has been considered to be the gold standard measurements of TrA performance which has been validated by imaging and electromyography. Previous studies with PBU demonstrated that individuals with low back pain have an impaired ability to depress the abdominal wall (Hodges et al., 1996). Hides et al. (2006) suggested that the TrA is important in sustaining the spinal cord and that its conditioning of TrA is accompanied by functional improvement.

The process of proprioception is based on all three stabilizing systems neural, muscular and ligamentous (Norris, 1995a, b). Jull et al. (1987) has reported that limbic stimulation and emotional stresses may adversely affect the feedback mechanisms and thereby resulting in poor motor control, disuse and affect a person’s ability to contract muscle consciously, which is consistent in our findings.

Research by Hodges et al. (1996) supports that TrA to have an anticipatory function, contracting prior to distal activity, consequently enhancing spinal stabilization. In addition, mechanics of selective contraction of the TrA decreases the laxity of the SI joint (Richardson et al., 2002) thus improving the stability of the joint thereby relieving pain and disability. As with par with the previous studies the ultimate goal of this study was to focus on normal automatic functioning of TrA, enhancing the stabilizing system which is achieved through PBU and conventional exercises. Our results confirm the hypothesis that PBU training can recruit the TrA.

When the transversus abdominis contracts, the intra-abdominal pressure (IAP) increases, and the tension of the thoracolumbar fascia increases (Panjabi, 1992; Ebenbichler, Oddsson, Kollmitzer, & Erim, 2001). Consequently, stabilization of the spine is maintained by the IAP in the abdominal cavity and the stiffness of the lumbar spine. Hides et al, 2006 found significant increase in the efficacy of TrA which has been thought to enhance the stability of the spine, and this is consistent with our result, suggesting that increased TrA muscle activity contributed to spinal stabilization.

To relieve and to avoid pain, women with PRPP adopt abnormal pattern of muscle activity, this abnormal pattern leads to unphysiological burden on muscles and joints which ends up in more pain which aggravate the dysfunction of muscles resulting in a vicious cycle (Pool-Goudzwaard, Vleeming, Stoeckart, Snijders, & Mens, 1998). Hence to reduce the development of abnormal patterns of muscle activity, training was aimed at affecting the dysfunction of the muscle- tendon- fascia system that controlled force closure of the pelvis is achieved, thus breaking the vicious cycle thereby reducing the pain and disability.

Our study showed that the pressure biofeedback training has significantly increases the TrA efficacy, while decreasing pain and disability. Our results confirm the hypothesis that PBU can recruit the TrA. Therefore, we suggest that PBU training with conventional exercises is useful in treatment of PRPP to prevent disability through selectively strengthening the TrA.

The study data demonstrated that the subject showed minimal improvement in pain, disability and TrA efficacy during the baseline phase and shown a steady improvement in all these variables during the intervention phase. It should be noted that findings of this study are limited because of single case study without controls.

Cautions must be taken when extrapolating the findings of this study to other populations. Therefore, further studies are warranted to assess the effect of PBU with more samples and a control group to determine the benefit and selective muscle facilitation associated with PBU training.

## 4. Conclusion

Impairment in muscle performance appears to be a feature in most of the PRPP. The results of this study suggest that core muscle performance can be retrained with pressure biofeedback stabilization training program in PRPP patients.

Physiotherapist should keep these options in mind when prescribing exercise for a patient with PRPP that demonstrates muscle impairment, but should apply caution to extrapolating the findings of this study to patients with multicausal low back pain and disability.

## 5. Clinical Relevance

The results of this single case study may provide useful information for physiotherapist to include pressure biofeedback training as an adjunct with an aim of treatment and prevention of chronic disability in rehabilitation of Pregnancy related low back pain patients.
